# Hidden in red: evidence for and against red camouflage in a jumping spider (*Saitis barbipes*)

**DOI:** 10.1007/s00114-024-01945-1

**Published:** 2024-10-16

**Authors:** Jasmin Laura Gerfen, Cynthia Tedore

**Affiliations:** Institute of Cell and Systems Biology of Animals, University of Hamburg, Martin-Luther-King Platz 3, 20146 Hamburg, Germany

**Keywords:** Crypsis, Visual signaling, Color vision, Achromatic vision, Salticidae, Freeze-drying

## Abstract

Investigating the conspicuousness of animal color patterns to different observers is crucial for understanding their function. This study examines the peculiar case of a jumping spider (*Saitis barbipes*) whose males display red and black ornaments during courtship despite an apparent inability to distinguish these colors. We propose that, through predator eyes, red may actually be a better match than black to the spiders’ leaf litter background, and that the black fringe of hairs surrounding red ornaments may blur with red at natural predator acuities and viewing distances to produce a background-matching desaturated red. In a field experiment, we test whether red ornaments reduce predation relative to red ornaments painted black, and find that, unexpectedly, spiders with red ornaments are more heavily predated upon. Having established birds as the spiders’ primary predators, we image the spiders in their natural habitat using an avian-vision camera. We find their red coloration to have similar color contrast, but lower achromatic contrast, with the background than black coloration. We also find that red and black elements blur together at typical avian acuities and viewing distances to produce lower chromatic and achromatic contrasts with the background than would be seen by animals with higher acuities and/or closer viewing distances. Interestingly, red ornaments appear orange or yellow when viewed obliquely, which reduces their achromatic, but not chromatic, contrast with the background. Our imaging results provide support for our hypothesis that red is camouflaging, whereas the results of our predation experiment do not. Any functional significance of the spiders’ red coloration therefore remains unresolved.

**Supplementary Information:**

The online version contains supplementary material available at 10.1007/s00114-024-01945-1.

## Introduction

Animals often face conflicting selective pressures between natural and sexual selection (Endler 1978). While being cryptic to predators and prey should often be favored by natural selection, being conspicuous to conspecifics is frequently important for signaling and, in that case, should be favored by sexual selection. Exploiting differences in observers’ visual systems is one way to avoid predation while still signaling to conspecifics. Some songbirds, for example, have evolved color patches less conspicuous to their predators’ visual systems than to their own; such patches consist of UV reflective or absorbent patches that generate more contrast in the UV range that songbirds are sensitive to than in the violet range that their predators are sensitive to (Håstad et al. 2005). Aposematic signals may also be tuned to be conspicuous to predators while remaining cryptic to prey. The highly toxic *Latrodectus* widow spiders, for example, display a red pattern against a black body, which is conspicuous to avian predators but invisible to insect prey (Brandley et al. 2016).

The effectiveness of camouflage depends not only on the color vision system of the observer but also on the combination of its viewing distance and visual acuity (Endler 1978; Gomez & Théry 2004; Penacchio et al. 2015). Thus, another way to signal to some individuals while remaining cryptic to others is to exploit differences in the typical viewing distances and/or acuities of different observers (Caves et al. 2018a, b). For example, some coral reef fishes have evolved contrasting stripes that are conspicuous to other coral reef fishes in close proximity but blur together to match the background for predator fishes that view the coral reef fishes from a larger distance (Marshall 2000; Marshall & Johnsen 2011). Similarly, the web stabilimenta (silk decorations) of some orb weavers may function to increase the visibility of the web to birds that would destroy the web if they flew through it; yet, stabilimenta have too fine of a spatial pattern to be visible to the majority of insect prey, which possess much lower spatial acuity (Bruce 2006; Caves et al. 2018a, b).

Here, we tackle the recently discovered evolutionary puzzle of the sexually dimorphic red ornamentation of *Saitis barbipes* jumping spiders. While females are a cryptic brown color, males are colorful, exhibiting red, black, and white patches on the thicker third pair of legs, and an orangish-red band over the eyes. The red coloration of the third pair of legs, which is vigorously waved during courtship, might until lately have been interpreted as a sexual signal. However, Glenszczyk et al. (2022) showed that *S. barbipes* lacks a red photoreceptor and is unlikely to be able to distinguish red from black, as red and black produce similar photoreceptor excitation patterns (Glenszczyk et al. 2022). Therefore, in the context of conspecific signaling, red and black coloration likely have a similar function of generating achromatic contrast with the background and with the spiders’ white color patches. Why *S. barbipes* have evolved both red and black ornaments rather than only black ones is unclear, but Glenszczyk et al. (2022) provided various alternative hypotheses that remain to be tested. One hypothesis argued that the black fringe of hairs surrounding the red leg patches might, at typical predator viewing distances, merge with the red patches to generate a perception of a brownish or dull reddish color, which might match the spiders’ normal substrate of leaf litter better than black alone would (Glenszczyk et al. 2022).

Potential predators of *S. barbipes* that should be able to discriminate red from black include birds and lizards, and their poor spatial acuity supports the above hypothesis. Birds have a median spatial acuity of 11 cycles per degree (cpd), with 84% of birds having acuity less than 30 cpd (Caves et al. 2018a, b; Land & Nilsson 2012). Spatial acuity in lizards is poorly known but ranges at least from 1.2 to 12 cpd (Fleishman et al. 2017; New & Bull 2011). These estimates contrast strongly with human spatial acuity, which is much higher at 73 cpd (Land & Nilsson 2012). As *S. barbipes* are very small (~ 4 mm), humans cannot easily distinguish between adjacent red and black patches on *S. barbipes* without the aid of assistive optics, and it is hard to imagine that the spiders’ natural predators, with their lower spatial acuity, would have this ability, either. To a human that has spotted a male *S. barbipes* in the forest, the legs do not look obviously red, but rather a dark achromatic color (pers. obs., JLG & CT). That said, even if a predator were able to resolve *S. barbipes*’ red and black patches, the spectral shape of leaf litter often resembles that of desaturated long-wavelength colors, i.e., yellows, oranges, and reds (Nagler et al. 2000; 2003). Thus, the spiders’ red coloration may, in fact, be a closer match to leaf litter than their black coloration is. Furthermore, the photoreceptors responsible for achromatic vision in birds and lizards peak in the yellow portion of the spectrum (Osorio 2019). Such photoreceptors can be expected to capture some photons from the rising portion of the red legs’ reflectance curve, weakening the achromatic contrast of what would otherwise be a very dark object against a lighter litter background (i.e., if the legs were completely black).

In this study, we aimed to test whether the red coloration of *S. barbipes* males (1) reduces their vulnerability to predators with a red photoreceptor class and (2) exhibits lower color and/or achromatic contrast with the natural background than black coloration would. For aim 1, naturally posed freeze-dried spiders were placed in the field. In the experimental group, the red patches on the third leg pair were covered with black paint. In the control group, the ventral, not visible side of the abdomen, was covered in black paint so that control spiders would produce the same chemical cues as experimental ones. The relative consumption of the two groups by predators was then documented throughout the day by camera traps. For aim 2, having established that birds are the dominant predators of *S. barbipes*, we imaged freshly killed spiders in their natural environment using an avian-vision multispectral camera. From these images, we calculated avian color and achromatic contrasts between the spiders’ red ornaments (i.e., red cuticle covered with red hairs) and the background and compared these to contrasts between a simulated black ornament (i.e., a combined selection of bare black cuticle and black hairs protruding from the margins of the metatarsus) and the background. The black ornament was simulated because *S. barbipes* lacks black cuticle covered with black hairs, and bare black cuticle exhibits UV iridescence, as does red cuticle, but this iridescence is largely hidden by red hairs on red cuticle (Glenszczyk et al. 2022). Contrasts between the red ornaments and the background were calculated using the camera’s native spatial resolution and the spatial resolution of the average bird from a distance of 30 cm to test whether the black fringe of hairs surrounding the red patches may blur with the red to make the red less discernible to predators foraging under natural viewing conditions. Both color and achromatic contrasts were calculated because birds are known to make use of both forms of contrast when searching for food (Stobbe et al. 2009). If our results suggest that red coloration does indeed serve as camouflage in these spiders, this could have broad implications for a variety of taxa whose high-chroma, long-wavelength-rich coloration has been assumed to function solely for signaling. This would be especially applicable to animals viewed against leaf litter and tree trunks, as these substrates are also rich in long wavelengths but have low chroma.

## Materials and methods

### Predation experiment

#### Preparation of spiders

For the predation experiment, 143 male *S. barbipes* were collected in a forest on the outskirts of Osp, Slovenia (45°34′34.7″N 13°51′23.0″E) over a span of 5 weeks between 10 May and 8 June 2022 and were used in experiments between 22 May and 26 June 2022. Males were housed separately in plastic containers with breathing holes before they were prepared for experiments. During this stage, they were sprayed with water every day. Individuals were housed in these conditions for up to 11 days. Spiders kept in vials for more than 1 week were fed 1–3 *Drosophila* spp. once. At least 24 h passed between feeding and killing.

The spiders used in the field study were freeze-dried to prevent them from rotting and to allow them to be used in more than one trial. The evening before freeze-drying, spiders were killed by overanesthesia with CO_2_ and kept in the refrigerator in an airtight container to prevent shriveling of the abdomen due to evaporation. The following morning, the dead spiders were positioned in lifelike positions on pieces of styrofoam with insect needles (without puncturing the cuticle). The positioned spiders were then flash frozen by placing them in a − 80 °C freezer for 1 h. The frozen spiders were then quickly transferred to a freeze-dryer with a vacuum of 0.220 mbar at − 25 °C (Christ Gamma 1–20 LMC-2, Osterode am Harz, Germany; prefreezing the chamber 2–5 h before start to − 40 °C, pump prepare 10 min) until completely dry, which took 3 to 4 days. After freeze-drying, the insect needles were removed, and the spiders stayed in the position they had been fixed in. To prevent breakage of legs and other body parts during subsequent storage and transport, dry spiders were stored on toothpicks inserted in styrofoam in a paper box. To attach the spiders to the toothpicks, the tip of the toothpick was cut off and the end covered in beeswax. The spider’s prosoma was then adhered to the toothpick with the wax.

In the experimental treatment, the red patches on the forward-facing surfaces of the tibia, patella, and femur of the third legs were covered with black paint. In the control treatment, the bottom of the abdomen was covered with black paint to ensure the spiders of the control group had the same chemical cues as the spiders of the treatment group. The paint was a mixture of chicken egg yolk and black liquid food coloring (V2 Foods, Tiefschwarz, Nordstemmen, Germany) with a ratio of 3 drops of food coloring per 1.0 g egg yolk. This paint composition was chosen due to its biodegradability and nontoxicity to birds. Spiders were painted under a stereo microscope using a small brush (Elita Miniatur Dreikantstiel Pinsel-Set: 10/0 Synthetik or Rotmarder-Kolinsky, Großhabersdorf, Germany). A larger brush (Elita Miniatur Dreikantstiel Pinsel-Set: 2/0 Rotmarder-Kolinsky, Großhabersdorf, Germany) was used to go over the painted spots once the paint started drying to slightly roughen the painted surface and reduce the shininess of the paint. When painting the spiders, as little paint as possible was used, while still covering the red patches. This was done to avoid changing the size of the legs due to paint application (Fig. 2a, b).

#### Validation of freeze-drying and painting techniques

**Fig. 1:**
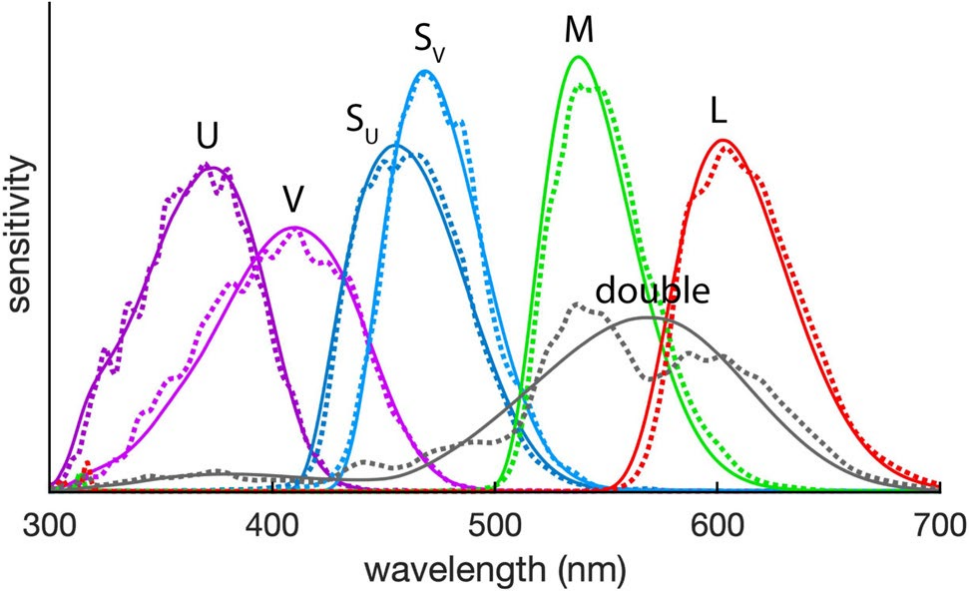
Target (solid) and effective (dotted) spectral sensitivities of modeled avian channels. Target sensitivities are those calculated from previously published physiological data and effective sensitivities are those of the multispectral imaging system (Tedore & Nilsson 2019). The average UVS bird possesses the U, S_U_, M, and L cones, whereas the average VS bird possesses the V, S_V_, M, and L cones, all of which contribute to color vision. Both visual systems possess similarly tuned double cones, which mediate achromatic vision. Curves are area normalized and do not reflect absolute sensitivities

Freeze-dried spider colors appeared very similar to those of live spiders, but to assess this quantitatively, five additional spiders were photographed before and after freeze-drying. All images were taken using the multispectral camera and six filters described in Tedore and Nilsson (2019), which were designed to enable the modeling of avian color vision. The camera consists of a monochrome sensor, a UV-transmissive lens mounted with an infrared blocking filter, and a filter wheel holding six filters, each custom-fabricated to mimic the spectral sensitivity of the average UVS and VS bird when combined with the other camera components (Fig. 1). Spiders were killed, freeze-dried, and positioned as experimental spiders, but the ventral side of the prosoma was glued to a nail. Each spider was photographed in front of a 20% reflective 2-inch fluorilon gray standard (Avian 194 Technologies, New London, NH, USA) before and after freeze-drying. To keep the spiders in the same position for both images, the insect needles were left in the foam. To maintain constant lighting conditions, images were taken under a xenon light source (XE-140BF, Seric Ltd., 196 Tokyo, Japan) closely mimicking the spectrum of natural daylight in an otherwise darkened room, as described in Glenszczyk et al. (2022).

Using custom code executed in MATLAB 2022b (Mathworks, Natick, MA, USA), dark noise was calculated by taking the mean value of several rows of camera pixels that receive no light and was subtracted from all pixels. Cone excitations were then calculated by first normalizing all pixels by the mean pixel value of the gray standard (i.e., converting to relative quantum catches *p*) and then using the $E = \frac{p}{p+1}$ nonlinear transformation described by Naka and Rushton (1966). For each spider, the red portion of one of the spider’s tibias was selected and its median cone excitation values compared in the before and after freeze-drying images. Areas occluded by shadows cast by the pins were omitted from these selections. Since the spiders’ legs often shifted position a bit during the freeze-drying process, and the angular position of the leg was found to affect color (see Results), we selected the tibia (left or right) that moved the least between the before and after freeze-drying images. One of the five spiders’ images had to be discarded because the non-ornamented walking legs were not positioned properly and occluded both red tibias.

To test whether the black paint appeared similar to a theoretical black cuticle covered with black hairs, a freeze-dried spider with painted legs was photographed with the same avian-vision camera described above. The spider was photographed within the field site situated in front of the same gray standard as above, and the images were processed in the same way. The black-painted areas were selected and their median cone excitations were calculated and compared to those of a selection combining the black metatarsal cuticle and dense clumps of black hairs projecting from the edges of the metatarsus. Since the red leg ornaments consist of red cuticle covered with red hairs, this seemed the most apt representation of what the spiders’ red legs would look like if they were black instead of red.

#### Experimental setup

Spiders were grouped into size-matched pairs, with one spider randomly selected to receive the experimental treatment and the other to receive the control treatment. Although in the forest, spiders were spaced several meters apart from each other, such that a predator could only encounter one spider at a time; this pairing ensured that one treatment did not consist of larger spiders than the other treatment. For size-matching, the prosoma width, span of the third legs in the freeze-dried position, and body length (head to abdomen) were measured to the nearest 0.5 mm under a stereomicroscope while positioned on millimeter paper. Treatment pairs were matched for span of the third legs, as other visual traits showed only small differences.

**Fig. 2:**
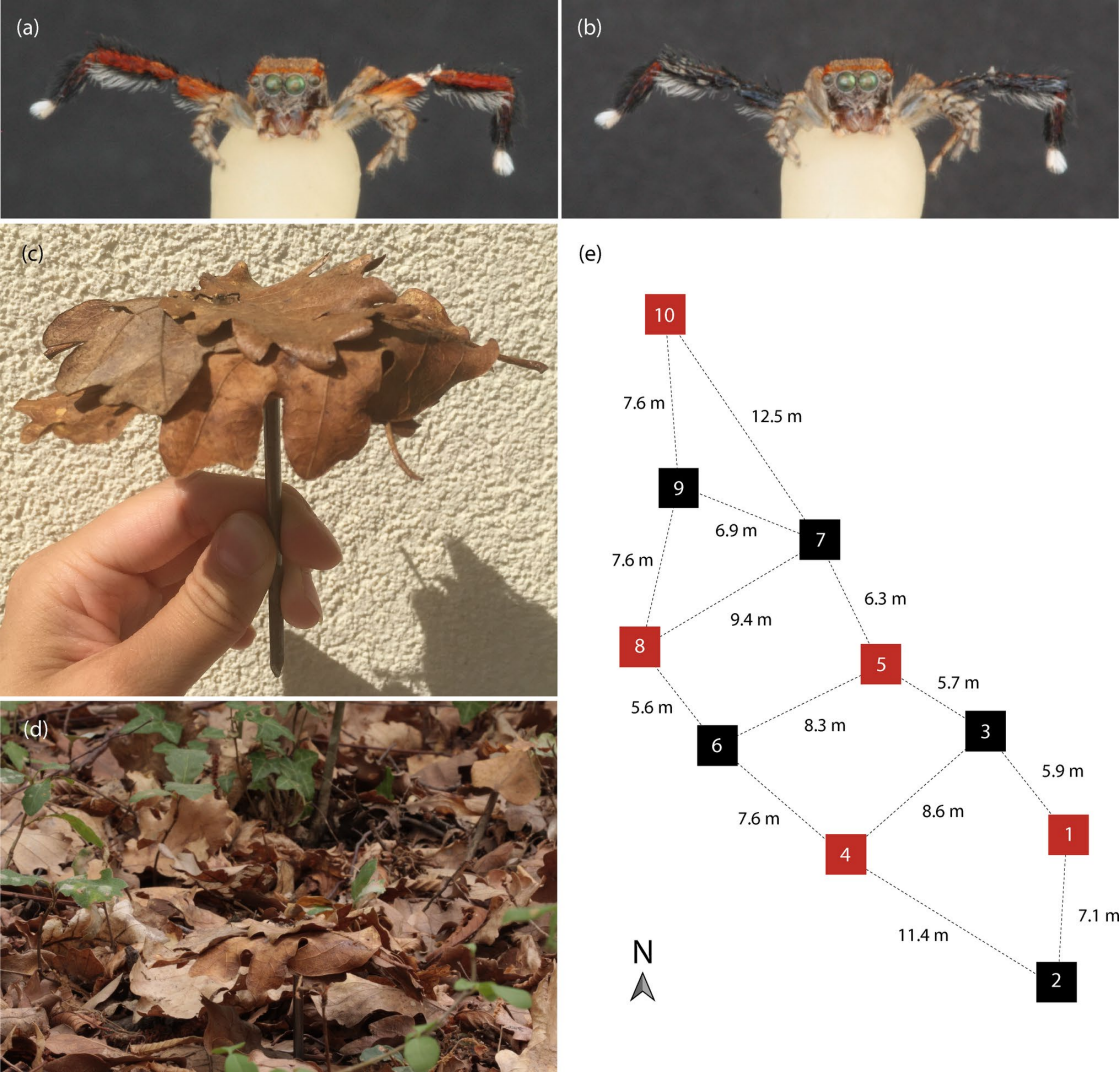
Summary of field experimental setup. Freeze-dried male S. *barbipes* before (**a**) and after (**b**) painting the femur, patella, and tibia of the third legs with black paint. **c**, **d** Exemplar platform with the leaf litter and freeze-dried spider attached to it before (**c**) and after (**d**) placement in the field. **e** Schematic illustration of the arrangement of the platforms within the study site, with platform colors indicating how treatments were alternated (with the opposite configuration used on consecutive days)

To present the spiders to predators, they were placed on little platforms raised slightly above the forest floor, with the platforms’ undersides smeared with petroleum jelly to prevent scavengers, like ants, from entering the platform from below and damaging the spiders. The platforms were made of 10-mm-thick pieces of wood (beech, 40 × 35 × 10 mm) with a nail (3.8 × 100 mm) inserted through the center. The head of the nail was recessed in the block to create a flat surface. The platform was covered with dead leaves taken from leaf litter found at the study site (Fig. 2c, d). As the leaf litter mainly consisted of oak leaves, only these leaves were used. Four leaves were chosen and glued on top of the platform, with the lower leaf surface facing downwards, each along one of the four sides, using hot glue. Leaves in this orientation naturally curled downwards, obscuring the edges of the platform. A fifth leaf was glued to the middle of the platform, on top of the previously described four leaves. It was randomly chosen whether the fifth leaf’s upper surface should face upwards or downwards, but for both spiders in a given treatment pair, the leaves faced in the same direction. Approximately the same number of pairs with upward- and downward-facing leaves were used over the course of the experiment (upward, 106 trials; downward, 116 trials). The spiders’ prosomas were attached to the top of the fifth leaf with a small amount of beeswax. Both the bottom of the platform and the undersides of the leaves sticking out beyond the edges of the platform were covered in petroleum jelly to deter scavengers. The nail protruding from the bottom of the platform was inserted into the ground to present the spiders slightly elevated above the forest floor such that none of the platform leaves touched the ground. This effectively prevented scavengers from crawling onto the platform. During all trials in which a spider was used, it remained on the same platform. Spiders were used in up to five trials if they were not damaged.

The chosen study site was an approximately 10 × 35 m area near the collection site (45°34′36.6″N 13°51′25.3″E). It was chosen because spiders and foraging birds were seen there before the experiment began and because the understory was relatively clear and the ground flat, which allowed the cameras to detect motion from a greater distance and with a broader field of view. Ten experimental locations were selected within the study site and used over the course of the experiment (Fig. 2e). Locations were at least 5 m apart to prevent birds from finding an inconspicuous spider only because a more salient spider was nearby. Each location was marked by placing an extra platform overnight between trials to ensure the same spots were used in all trials.

At each of the 10 experimental locations, dried mealworms (Deli Nature Greenline Mealworms, Schoten, Belgium) and a seed mix (Versele-Laga Prestige Parrots, Deinze, Belgium) were dispersed to attract predators to the site. To avoid attracting boars, which would trample the experimental setup and scare away smaller predators, the mealworms were tossed in chili powder (2.5 g chili powder per 100 g dried mealworms). This should have made them less attractive to mammals at the site, which lack red photoreceptors, while not repelling birds (Baylis et al. 2012). Larger pieces of seeds, like nuts and big corn pieces, were taken out of the seed mixture to prevent larger food particles from damaging the gastrointestinal system of young birds. To standardize food dispersal, the same amount of food (5 g mealworms; 14 g seed mix) was dispersed at every location with a self-made “food sprinkler,” which consisted of a jar with a lid of 7 cm diameter punctured with six evenly spaced holes. The designated amount of food was put in the food sprinkler, which was shaken lightly, directly above the platform location at a consistent height (1.5 m), until all food was released. Seeds and mealworms were dispersed separately.

To further attract predators, a beige clay water bowl (ø 19 cm) was placed about half a meter in front of each spider and refilled with fresh water before every trial. A trail camera (T100 Trail Camera, Campark, Hong Kong, China) with a camouflage pattern on it was placed behind each spider, facing the platform, so that the camera, the platform, and the water bowl formed a line. The placement of the water bowl in front of the spider was intended to bias birds to approach the spider from the front because the spiders’ red coloration is most visible from this angle. The cameras took videos of 30 s in 4 K resolution at 30 frames/s every time the sensor was activated with a trigger distance of up to 20 m. The camera uses passive infrared (PIR) sensors that activate the camera when temperature changes are detected. The PIR Interval was set to the camera’s minimum value of 10 s to avoid missing any actions of predators. PIR sensitivity was set to “High.” Side PIR was turned on, meaning that lateral PIR sensors were activated, creating a total induction angle of 120°, to detect potential predators as early as possible.

The marking platforms, which were left overnight and assured the recovery of the experimental locations, were replaced by the prepared platforms according to the beforehand randomized locations of the different spiders. To ensure no scavengers could climb onto them, platforms were positioned just high enough to ensure that none of the leaves glued to the platform touched the ground. The spiders were positioned randomly facing north, south, east, or west. The size-matched treatment pairs were always placed at adjacent sites (Fig. 2e; locations 1 and 2, 3 and 4, etc.), and among pairs, treatment colors were spatially alternated to prevent clumped finding of one treatment by predators. Each day, the arrangement of treatment and control spiders was alternated so that the same treatment was not at the same location on two consecutive days. This was done to prevent predators from getting used to a treatment at a given location and to rule out any influence of varying popularity of the different locations. Furthermore, although there were not always ten spiders placed at the study site, due to a lack of enough prepared spiders on some days, there was always the same number of experimental and control spiders at the study site. This ensured the predators’ choices were not biased by the number of encounters with different treatments.

Trials started after the setup was completed, which was around seven o’clock in the morning. Experiments went on for 12 h, during which time the study site was not entered to avoid disturbing potential predators. The hours of the day during which experiments were conducted were chosen to secure photopic lighting conditions. Since only three people were seen near the study site over the course of the whole experiment, and none of them were in the study site or on the hiking path nearby, predators were most likely not disturbed by humans. On extremely windy days (≥ 50 km/h), no experiments were performed because most spiders would have been taken by ants, as falling tree parts created bridges that enabled them to enter the platforms. In total, 222 trials were performed on 24 experimental days.

All recorded videos were checked for the appearance of potential predators. Trials in which no potential predator was filmed over the duration of 12 h were excluded from analysis, as the spiders used in these trials were not exposed to the risk of being eaten. Furthermore, trials in which potential predators were filmed but were not foraging close to the ground or interacting with the supplied water (e.g., flying or running through the frame) were excluded, as spiders in these trials were also thought to be exposed to a minimal risk of predation. For the remaining videos, it was checked whether the spiders were eaten and, if so, how much time passed from the beginning of the trial to the predation event (i.e., the survival time), and which predator species preyed on the spider. Trials in which the spider was damaged, but no predation event was caught on camera, were excluded since it was unclear when they were damaged and whether the damage was inflicted by a predator with red vision. To get an overview of the predators with red vision at the study site, all such taxa caught on camera during trials were noted.

#### Statistical analysis

To analyze the spiders’ survival probability, a mixed-effects Cox proportional hazards model (Cox 1972) was run in R (R Core Team 2022) using the “coxme” package (Therneau 2022a). A spider being eaten by a predator represented an event and a trial in which the spider was not eaten was treated as censored. The survival time was rounded to the nearest minute. The fixed effect was the color of the legs and the random effects were spider ID and date (to control for weather and lighting conditions). The size of the spiders (span of the third legs) was ignored, as there was no significant difference between the size of spiders across treatments (Mann Whitney *U* = 1796.5, *N*_black_ = 59, *N*_red_ = 56, *p* = 0.406, median span of third legs: red = 9.0 mm, black = 9.0 mm). The “cox.zph” function of the “survival” package (Therneau 2022b) showed the proportional hazard assumption of the Cox model to be met.

### Measurements of contrast with the background

#### Preparation of spiders and multispectral imaging

Between 22 and 28 June 2022, 13 male *S. barbipes* were collected, anesthetized in the field with CO_2_, and posed on dead leaves in the approximate location where they were collected (not on the platforms used in the predation experiment above). Most individuals were photographed on three different leaves, although some were photographed on only 1–2 leaves if the spider became damaged or its legs began curling before all photos were taken. In total, spiders were photographed on 34 different leaves. Leaves were randomly selected with the researcher sitting on the ground and reaching behind herself to blindly pick up a leaf out of her field of view. The side of the leaf that was first visible when the leaf was brought into the researcher’s field of view was the side the spider was positioned on.

The initial goal was to photograph spiders facing the camera with the ornamented third pair of legs positioned perpendicular to the direction of view of the camera, which is the approximate angle at which they are presented during courtship. Early on, however, it was discovered that the legs’ red coloration appeared orange or even yellow when angled away from the camera. To quantify this effect, some individuals were therefore photographed in two different positions: one facing the camera and one with one or more legs angled away from the camera at roughly 45° (approximated by eye). Later, we boosted our efficiency by instead taking a single photo with one leg oriented perpendicular to the camera and one leg angled 45° away. In total, 13 spiders were photographed, seven of which were photographed with legs in both perpendicular and oblique orientations. The spiders took up a large portion of the image frame such that the span of the posed ornamented pair of legs took up at least one-quarter of the width of all images.

Posed spiders were photographed using the same avian-vision multispectral camera as described above, using the same six single-cone filters and two additional filters previously used in Glenszczyk et al. (2022), which enabled us to generate a computational double-cone filter mimicking the avian double cone, which is responsible for achromatic vision (Fig. 1; for an explanation of computational filters, see Tedore & Nilsson 2021; Glenszczyk et al. 2022; Tedore 2024). The angle of the camera mimicked the viewpoint of a bird foraging on the ground, ranging from looking horizontally at 0° to looking down at 45° and anywhere in between.

As above, dark noise was subtracted from all pixel values. Chromatic adaptation of the eye to the background was simulated in each channel by dividing each pixel by the mean value across all pixels. These values were then converted to nonlinear receptor excitation values using the same $E = \frac{p}{p+1}$ transformation used above (Naka & Rushton 1966).

The entire background (excluding the spider) and different sections of the third pair of legs were then selected for analysis using MATLAB’s “roipoly” tool. The entire red (or orange or yellow, depending on the viewing angle) patch on the patella and tibia was selected and its median cone excitations calculated. As this patch includes red hairs against a red cuticle, for equivalency, we selected the bare black cuticle on the metatarsus and aimed to select a roughly equal area of dense black hairs projecting from the edges of the metatarsus, taking care not to select any background pixels, and calculated these pixels’ median cone excitations. By combining black cuticle and black hairs into a single selection, we approximated the color of a theoretical black tibia and patella covered with black hairs. Since black and red cuticle both exhibit UV iridescence, and the red hairs on the red cuticle largely mask this iridescence, selecting the bare black cuticle alone would not have accurately represented what black cuticle covered with black hairs would look like. Exemplar images showing the patches selected can be seen in Supplementary Fig. 1.

Since all birds predating *S. barbipes* and most birds at the site likely possess the UVS visual system (see Results), we limited our analyses to the UVS visual system. Color and achromatic contrasts were calculated using the receptor noise-limited model (Olsson et al. 2018; Vorobyev & Osorio 1998). Cone ratios used were those reported by Hart (2001) for the blue tit (1:1.9:2.7:2.7 U:S:M:L), a close relative of the great tit, which was the species that predated the majority of the spiders in this experiment (see Results). For color contrasts, noise in a single photoreceptor was estimated to be 0.1, and for achromatic contrasts, 0.34; both estimates were based on passerines in which these values have been measured (*Leiothrix lutea* and *Taeniopygia guttata* for color contrasts and *Sturnus vulgaris* for achromatic contrasts) (Olsson et al. 2018).

Color and achromatic contrasts were calculated between 20 ± 3 evenly-spaced programmatically-selected (i.e., automatically selected using code) patches of 3 × 3 pixels in the background and (1) the red tibia + patella oriented perpendicular to the camera, (2) the red tibia + patella angled away from the camera, and (3) the theoretical black tibia and patella (consisting of a combined selection of black metatarsal cuticle and hairs oriented perpendicular to the camera). Calculations 1–2 were conducted once using the camera’s native spatial resolution and once after blurring the images to mimic the resolution of the average bird (11 cycles per degree) with a viewing distance of 30 cm. This blurring was achieved with custom MATLAB code using the method described in Caves and Johnsen (2018).

#### Statistical analysis

Differences in color and achromatic contrasts were evaluated between 1 and 2 above at both the camera’s native spatial resolution and that of the simulated predator (testing the fixed effects of viewing angle and spatial resolution) and between 1 and 3 above (testing the fixed effect of red versus black coloration). Due to unequal variances across different fixed and random effect levels, we used the “robustlmm” R package for robust estimation of linear mixed-effects models. Spider ID and leaf ID were initially included as random intercepts in all models, since spiders were photographed against 1–3 different leaves and 20 ± 3 patches were sampled from each background. We attempted to fit random slopes of each fixed effect over each random effect, but some random slopes had to be removed due to nonsingular fits or perfect correlation with random intercepts. Random intercepts corresponding to spider ID were also removed because their variance was consistently zero. The fixed and random effects in all final models can be seen in Table 1.

**Table 1:** Summary of robust linear mixed-effects models (measurements of contrast with the background)

Model	Dependent variable	Fixed effects	Coeff	95% CI	Random effects	Leaf *N*	Spider *N*
1	Color contrast	Color (red)	− 0.066	[− 0.25, 0.12]	(color|leaf)	34	13
2	Color contrast	Angle (perpendicular)	− 0.0050	[− 0.081, 0.072]	(angle|leaf)	17	7
		Acuity (predator)	** − 0.46**	[− 0.49, − 0.44]		34	13
3	Achromatic contrast	Color (red)	** − 0.16**	[− 0.19, − 0.13]	(1|leaf)	34	13
4	Achromatic contrast	Angle (perpendicular)	**0.099**	[0.053, 0.14]	(angle|leaf)	17	7
		Acuity (predator)	** − 0.16**	[− 0.18, − 0.14]		34	13

Coefficients printed in bold are significantly different from zero

Since the black-painted legs evoked somewhat greater cone excitations than the black metatarsus (see Results), we ran the above color and achromatic analyses a second time, this time artificially boosting the black metatarsal cone excitations to determine whether our predation experiment’s results were likely to have been affected by this discrepancy in apparent brightness.

## Results

### Predation experiment

**Fig. 3:**
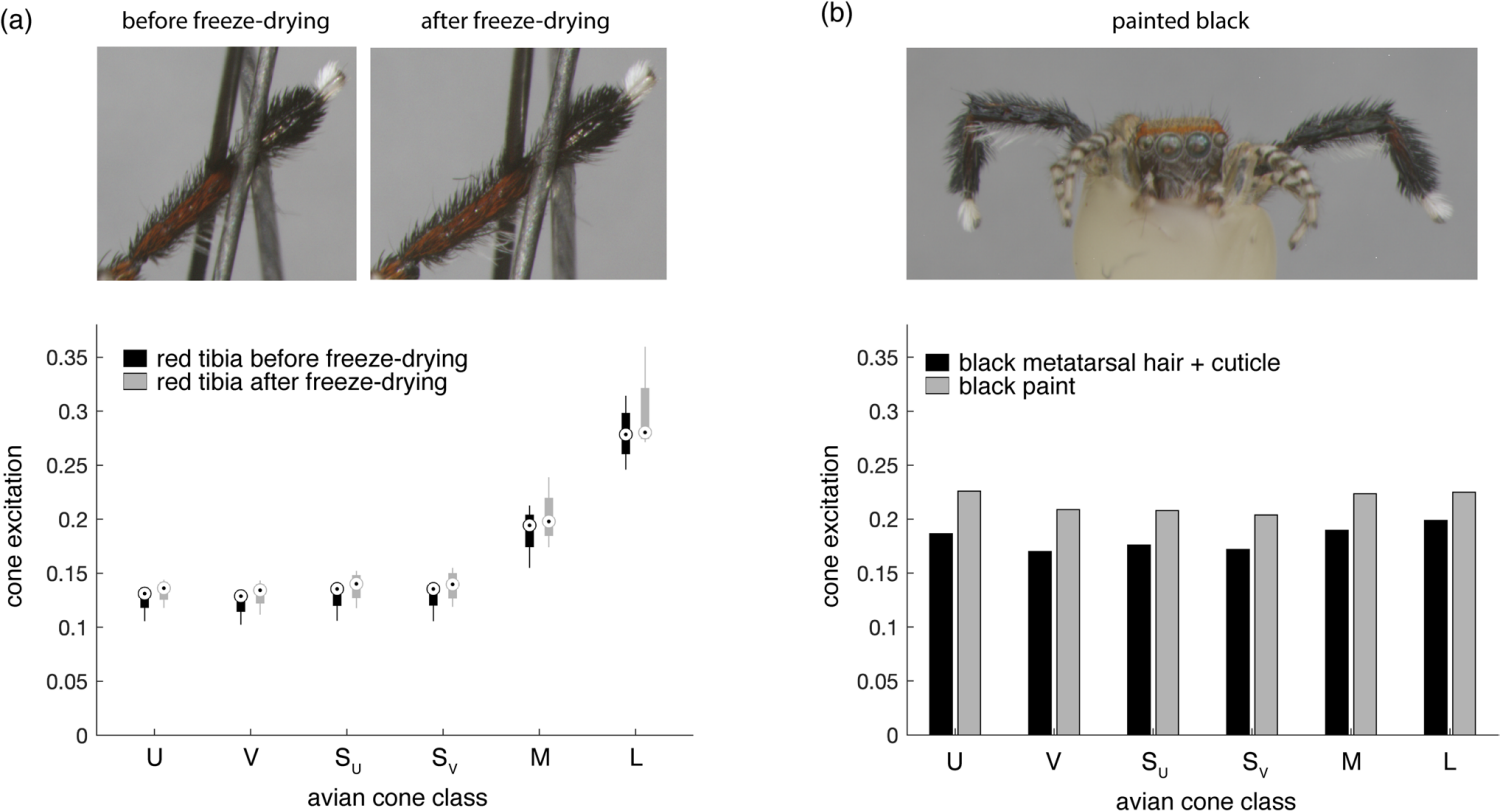
Assessment of the freeze-drying (**a**) and painting (**b**) techniques. **a** Comparison of the cone excitations induced in the UVS and VS avian visual systems by *S. barbipes*’ red tibia before and after freeze-drying (*N* = 4), including exemplar false-color photos of one individual’s leg before and after freeze-drying. Plotted data are boxplots with the median, interquartile range, and maximum and minimum values shown. **b** Comparison of the cone excitations induced in the UVS and VS avian visual systems by *S. barbipes*’ black metatarsus and the black paint used in the predation experiment (*N* = 1), including a false-color photo of the individual measured. In all false-color images, avian L cone excitations are plugged into the R channel of the computer display, M excitations into the G channel, and S_U_ excitations into the B channel. Note that the absolute cone excitation values of the red and black patches in (**a**) and (**b**) should not be compared, as the spiders were photographed under different lighting conditions

The comparison of spiders before and after freeze-drying showed that, across all cone classes, cone excitation in response to the spiders’ red tibia differed by a mean of 6.9% (std = 4.7%) (Fig. 3a). In most cases, cone excitations were slightly higher after freeze-drying than before. The comparison of the black-painted legs to the simulated black ornament (combined selection of metatarsal cuticle and hairs) showed the two colors to be similar in spectral shape (roughly achromatic), but with the black paint appearing a mean of 18.6% (std = 3.3%) brighter (Fig. 3b). As the diffuse reflectance of the paint was very low, around 1% (data not shown), the higher reflectivity of the paint under natural conditions appears to be due to specular reflection of the surrounding environment, which can be seen in the false-color image in Fig. 3b.

Bird species that were caught on camera and could be identified included great tits (*Parus major*; 4326 videos), European robins (*Erithacus rubecula*; 96 videos), Eurasian blue tits (*Cyanistes caeruleus*; 53 videos), blackbirds (*Turdus merula*; 42 videos), common chaffinchs (*Fringilla coelebs*; 10 videos), hawfinches (*Coccothraustes coccothraustes*; 6 videos), Eurasian nuthatches (*Sitta europaea*; 4 videos), great spotted woodpeckers (*Dendrocopos major*; 3 videos), and a treecreeper (*Certhia* spp.; 1 video). All but woodpeckers are known (or likely, based on phylogeny) to possess the UVS visual system (Ödeen et al. 2011). Aside from one lizard (1 video), no other predators with red vision were caught on camera. Lizards in general seemed to be uncommon in *S. barbipes* habitat, as the authors did not spot any by eye over the course of specimen collection and experimentation.

**Fig. 4:**
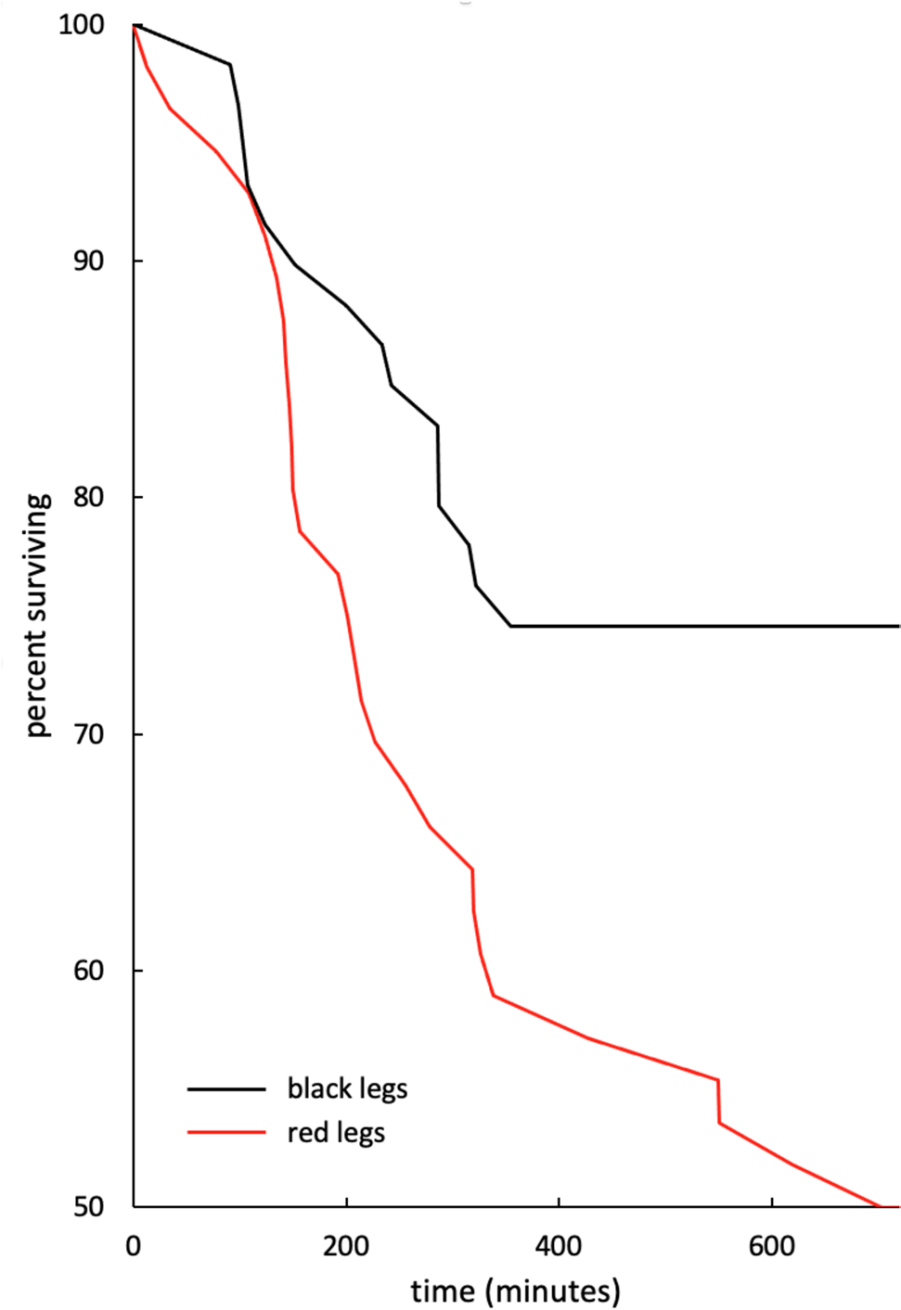
Percentages of *S. barbipes* males with painted black legs versus unpainted red legs surviving over the course of 12 h

In total, 115 out of 222 trials were included in the analysis, i.e., 59 trials including a spider with painted black legs and 56 trials including spiders with unpainted red legs (leaf upwards, 57 trials; leaf downwards, 58 trials). A total of 4450 videos of potential predators were taken during these trials. Out of 115 trials, 43 spiders were eaten by birds, including 15 spiders with painted black legs and 28 spiders with unpainted red legs (see Suppl. Videos 1–2 for exemplar videos of predation events). Of those, 42 spiders were eaten by great tits (*Parus major*), of which at least 39 were eaten by juveniles, and one spider was eaten by a common chaffinch (*Fringilla coelebs*). Leg coloration significantly influenced survival probability: having black legs reduced the hazard of being eaten by 63% (coefficient =  − 0.982; standard error = 0.411; Wald statistic =  − 2.39; *p* = 0.017) (Fig. 4).

### Measurements of contrast with the background

**Fig. 5:**
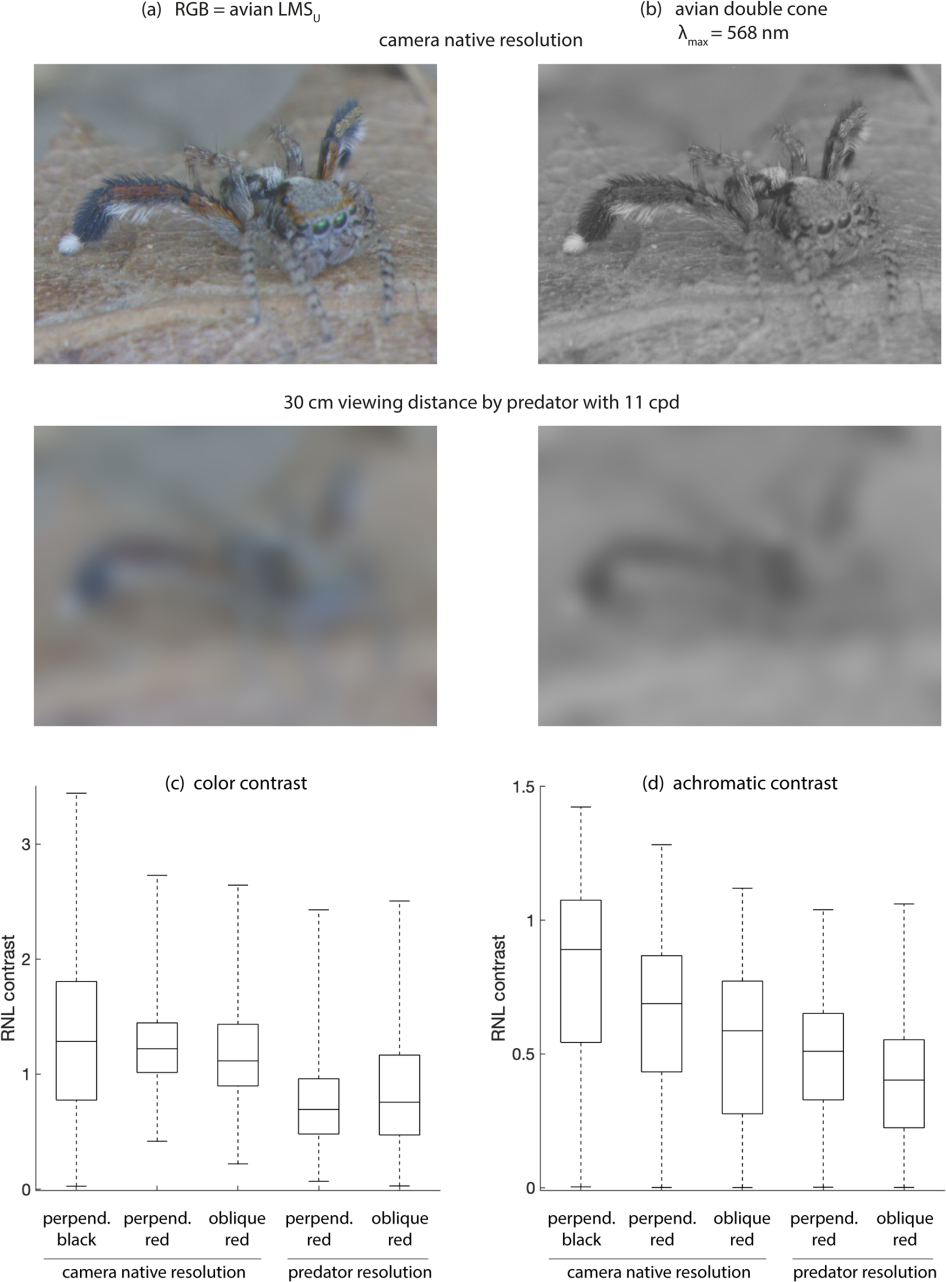
*S. barbipes*’ visual contrast with the background through UVS bird eyes. **a** Exemplar false-color receptor excitation images of a male *S. barbipes* with its right leg perpendicular to the camera and its left leg angled away. Avian L, M, and S_U_ cones are plugged into the R, G, and B phosphors of the computer display. **b** The same spider viewed through the achromatic (double cone) channel of birds. In both (**a**) and (**b**), the top image shows the spider at the camera’s native resolution and the bottom image shows it with the spatial resolution of an average bird (11 cycles per degree) viewing the spider from a distance of 30 cm. Note that the patella and tibia appear red and darker than the background when oriented perpendicular to the camera and orangish-yellow and closer in brightness to the background when angled away. Photos have been cropped from their original size. **c** RNL color contrast and **d** RNL achromatic contrast with the background for the red patella + tibia and the simulated black ornament of the perpendicularly oriented third leg and for the red patella + tibia of the obliquely oriented third leg at camera native and predator spatial resolutions. Boxplot whiskers represent maximum and minimum values. For perpendicularly-oriented body parts, spider *N* = 13, whereas for obliquely oriented body parts, spider *N* = 7. Each spider was photographed against 1–3 different leaves (for a total of 34 leaves for perpendicularly oriented body parts and 17 for obliquely oriented body parts), and contrasts were calculated between body parts and 20 ± 3 background patches in each image. See Table 1 for associated statistics and Suppl. Figure 2 for individual boxplots of each unique combination of spider and leaf

Red and simulated black coloration did not significantly differ in their color contrast with the background. However, achromatic contrasts with the background were significantly lower for the red coloration than for the simulated black coloration (Fig. 5, Table 1). This remained true when the cone excitations evoked by the simulated black coloration were artificially boosted by 18.6% to match those evoked by the black paint used in the predation experiment. Indeed, the direction of the effects remained the same (and significant) even with an increase in cone excitations of up to 29%. Thus, the greater brightness of the black paint is unlikely to serve as an explanation for the opposing results of the predation experiment. Color and achromatic contrasts with the background were also significantly lower for the spiders’ tibia + patella when viewed at the spatial resolution of the simulated avian predator than when viewed at the camera’s native resolution. This was true not only for the viewing distance of 30 cm shown in Table 1 and Fig. 5 but also for viewing distances of 20 and 40 cm. However, at closer viewing distances of 5 and 10 cm, this was only true for chromatic contrasts (results not shown). Finally, an oblique viewing angle lowered achromatic, but not color, contrasts of the red coloration with the background (Table 1; Fig. 5).

## Discussion

In this study, we tested the influence of male *S. barbipes*’ red coloration on their vulnerability to predators with red vision. The field experiment revealed a survival benefit of males with manipulated (painted black) leg coloration, which contradicted our hypothesis that red is camouflaging. By contrast, multispectral imaging and visual modeling indicated red to be a closer achromatic match, and a similar chromatic match, to the background as black coloration, which supported our hypothesis that red is camouflaging. Which result should be considered more reliable is unclear, as both methods had potential limitations (discussed below). Multispectral imaging also revealed that the redness of the legs is angle-dependent, turning from red when viewed perpendicularly to orange or yellow when viewed obliquely. This has the effect of lowering the red ornaments’ achromatic contrast with the background when viewed obliquely. This angle-dependency surprised us, as we had not noticed it despite spending considerable time viewing the spiders through a dissecting microscope. This emphasizes the importance of examining and quantifying animal color patterns from a variety of viewpoints (as recently articulated by Stuart-Fox et al. (2020)) in order to better understand their potential (multi-)functional significance.

Both the predation experiment and the visual modeling had potential limitations which makes it difficult to assess which result should carry more weight. The contradictory results are a useful reminder that most experiments have at least some limitations, and unless multiple different approaches point to the same answer, one should treat any one result with caution. One limitation of visual modeling, for example, is that the opponent comparisons made by birds are poorly known (but see Osorio et al. 1999). It is standard to include all possible opponent comparisons when calculating color contrasts; however, including a subset of them can be expected to have an effect on output. It is possible that, if the true opponent comparisons made by great tits were known, the result regarding color contrast could have been different (although the result regarding achromatic contrast would remain the same).

Another limitation of visual modeling is that it does not account for various cognitive effects on detectability. Some species, for example, have been shown to exhibit categorical color perception, i.e., they sort continuously varying wavelengths into discrete categories, much as humans divide the visible spectrum into colors we call red, yellow, green, etc. (Skelton et al. 2017). Zebra finches (*Taeniopygia guttata*) were recently shown to discriminate more sharply between certain colors than others, despite being similar distances apart in modeled color space (Caves et al. 2018a, b; Zipple et al. 2019). In the present study’s field experiment, if red were perceived as categorically quite distinct from the brown and other near-achromatic colors characteristic of leaf litter, then the spiders’ red legs could attain greater saliency due to the so-called pop-out effect. The pop-out effect describes the “popping out” of a target defined by a unique visual property (e.g., wavelength composition) of a scene, regardless of the number of distracting objects in the background (Healey & Enns 2012). This effect has been demonstrated across disparate vertebrate taxa, from primates to birds to fish, suggesting that this form of preattentive parallel analysis of the visual field might be a universal mechanism across vertebrate visual systems (Ben-Tov et al. 2015; Orlowski et al. 2015). Another limitation to our modeling approach is that it does not account for how the brain categorizes objects. Black objects may be more likely to be automatically categorized as shadows or cracks in the dead leaves the spiders stand on, as the color of very dark shadows is not discriminable from black. Research on humans has shown that shadows are processed automatically and not consciously noticed or remembered (Casati & Cavanagh 2019); thus, a black spider leg mistakenly categorized as a shadow may be automatically discounted and ignored. Although none of these potential cognitive effects could be accounted for in the visual modeling, they should have been accounted for in the field experiment and may explain the contradictory results obtained by the two methodological approaches.

One limitation of the field experiment, however, is that it made use of predators that were likely not naïve to unmanipulated *S. barbipes* with red legs, as such spiders are abundant in their habitat. Predators may therefore have already developed a search image for spiders with red legs. The use of search images, a selective attention to specific features of prey, has been demonstrated in various bird species (Bond 1983; Bond & Riley 1991; Dawkins 1971; Langley 1996; Lawrence 1985; Pietrewicz & Kamil 1979), although their use by foraging great tits is controversial (Lawrence 1986; Royama 1970; Smith & Dawkins 1971). That said, most spiders were predated on by juvenile birds, which may not have gained sufficient foraging experience to develop a search image for these spiders. To eliminate the possibility of a pre-existing search image for *S. barbipes*, this experiment could be repeated in another forest with similar environmental conditions and a similar bird population, but where *S. barbipes* does not occur.

Even if the resident birds in the field site did not have a search image of *S. barbipes*, their co-occurrence and the spiders’ abundance may have led to a certain familiarity with the normal red phenotype, and by contrast, neophobia for the manipulated black phenotype. Neophobia is well known in birds (Greenberg & Mettke-Hofmann 2001) and may have led birds to reject spiders with black legs, as such spiders may have been perceived as novel stimuli, inducing a sensation of uncertainty or even fear. That said, birds were only in rare cases observed to actively fixate on a spider without consuming it afterwards. This makes the neophobia explanation less likely, although we cannot rule out the possibility that birds perceived the presence of prey without obviously fixating on it. However, most spiders were predated on by juvenile birds, which are expected to show neophilic, rather than neophobic, behavior (Greenberg & Mettke-Hofmann 2001; Heinrich 1995). For juveniles, neophilia has greater advantages than for adults, as they come into a fully unknown environment lacking any experiences. This increases the potential benefits of exploring in comparison to adults (Greenberg & Mettke-Hofmann 2001). Young animals might also still be under parental protection, decreasing potential risks of general neophilia (Greenberg & Mettke-Hofmann 2001). Indeed, in a comparative study of different European tit species, Exnerová et al. (2007) did not observe neophobic behavior in juvenile great tits. Unlike adults, juvenile birds accepted aposematic prey when first presented to them. Food neophobia was also shown to be low in adult great tits of two distinct European populations (Exnerová et al. 2015). Hence, neophobia is unlikely to be the explanation for the lower predation rate of manipulated black spiders, as most spiders were eaten by great tits.

Another limitation of the field study is uncertainty about predator sample size. As birds could only be discriminated according to species and sex, it is unclear how many unique individuals predated on *S. barbipes* in this study. The 43 eaten spiders could have been preyed upon by 3 to 43 individuals (although at least seven unique individuals appeared in the videos; i.e., one common chaffinch and four juvenile and two adult great tits). If on the lower end, a few atypical individuals could have skewed the results in a direction unrepresentative of the population as a whole. It is also unclear whether great tits are the most important predators of *S. barbipes*. It could be that the seeds and mealworms used to attract birds to the platforms attracted species that normally exert lower predation pressure on *S. barbipes* than other species. Even if great tits were *S. barbipes*’ most important predators in the population we studied, this may not be the case across the rest of *S. barbipes*’ range, which extends across much of southern Europe. Finally, the method of presentation may not have been well suited to the foraging styles of some species. For example, the elevated platforms may not have been conducive to the foraging style of birds that find invertebrates by digging through leaf litter, or to small lizards, which may not have been able to easily see or reach the spiders on the platforms. A study of stomach contents of resident birds and lizards would probably be the most definitive method to determine the most important predators of *S. barbipes* with red vision.

If we imagine for the moment that the results of the predation experiment are the ones to be trusted, then red coloration would seem to have a survival cost. If this is the case, then it would be interesting to investigate why the combined forces of natural and sexual selection have not replaced red with black coloration. As proposed by Glenzczyk et al. (2022), red could be worth the cost of increased visibility to predators if the greater reflectivity of red coloration, as compared to black, was to reduce the build-up of heat in body tissues. This could impart a survival advantage to *S. barbipes* males as they live in a climate that regularly surpasses 30 °C towards the latter half of their breeding season. *Saitis barbipes* favor shady habitats and are difficult to find close to midday (pers. obs., JLG & CT), which suggests that they are indeed vulnerable to high temperatures and have behavioral adaptations to avoid them. It would be interesting to test for a cline in males’ red coloration across *S. barbipes*’ range. If populations living in hotter regions were brighter red or had a greater coverage of red coloration than populations living in cooler regions, this would provide some support for the idea that males’ red coloration has evolved to enable them to signal effectively to females while dissipating heat more effectively than would be possible if their red coloration was black (Glenszczyk et al. 2022).

Finally, it is worth noting that the color contrast of black coloration was much more variable than that of red coloration, i.e., sometimes, black coloration exhibited much greater and sometimes much lower color contrast with the background than red coloration did (Suppl. Figure 2a). This variability suggests that in another forest, with a slightly different color palette of litter on the forest floor, red could, on average, be camouflaging. It would therefore be useful to repeat the imaging experiment in other parts of *S. barbipes* range.

While we were unable to determine why *S. barbipes* males have evolved red leg coloration, our study is the first to test how freeze-drying affects arthropod coloration and to determine that freeze-dried specimens appear similar enough to live specimens to be used for predation experiments. Given the challenges of generating artificial prey that appear exactly like real prey, especially when angle-dependent coloration is involved, using freeze-dried arthropods provides a valuable method for testing the effect of animal coloration on the detection and survival of prey. Our study is also the first, to our knowledge, to document salticid predation by parid and fringillid birds. Previous studies of predation threats on jumping spiders have focused on predation by spiders (Harland & Jackson 2000; Huang et al. 2011; Su & Li 2006) and ants (Nelson et al. 2004) or have relied on manipulation of bird abundance (Mooney 2006, 2007; Mooney & Linhart 2006) or analysis of bird stomach contents and food delivered to nestlings (Billermann et al. 2022; Buainain & Forcato 2016; Davis & Arnold 1972; Fogarty & Hetrick 1973; Glutz von Blotzheim & Bauer 1987; Stiles 1995; Ueng et al. 2009) to determine whether birds predate jumping spiders. Our study is the first to provide direct video documentation of birds feeding on jumping spiders, and more specifically, to document predators of *S. barbipes*.

## Supplementary Information

Below is the link to the electronic supplementary material.

Supplementary file1 (PDF 2576 KB)

Supplementary file2 (MP4 119311 KB)

Supplementary file3 (MP4 119061 KB)

## Data Availability

With the exception of camera trap videos (504 GB), all data and code used for processing and analysis are available at https://figshare.com/s/96f18b869a765bb543df. Camera trap videos can be provided upon request.
