# Supplementary material for: Hidden in red: evidence for and against red camouflage in a jumping spider (Saitis barbipes)
Source: Naturwissenschaften. 2024 Oct 16;111(6):58. doi: 10.1007/s00114-024-01945-1 (PMC11485036; doi:10.1007/s00114-024-01945-1)
Supplement: Supplementary file 1 — Supplementary file1 (PDF 2576 KB) [file 114_2024_1945_MOESM1_ESM.pdf]

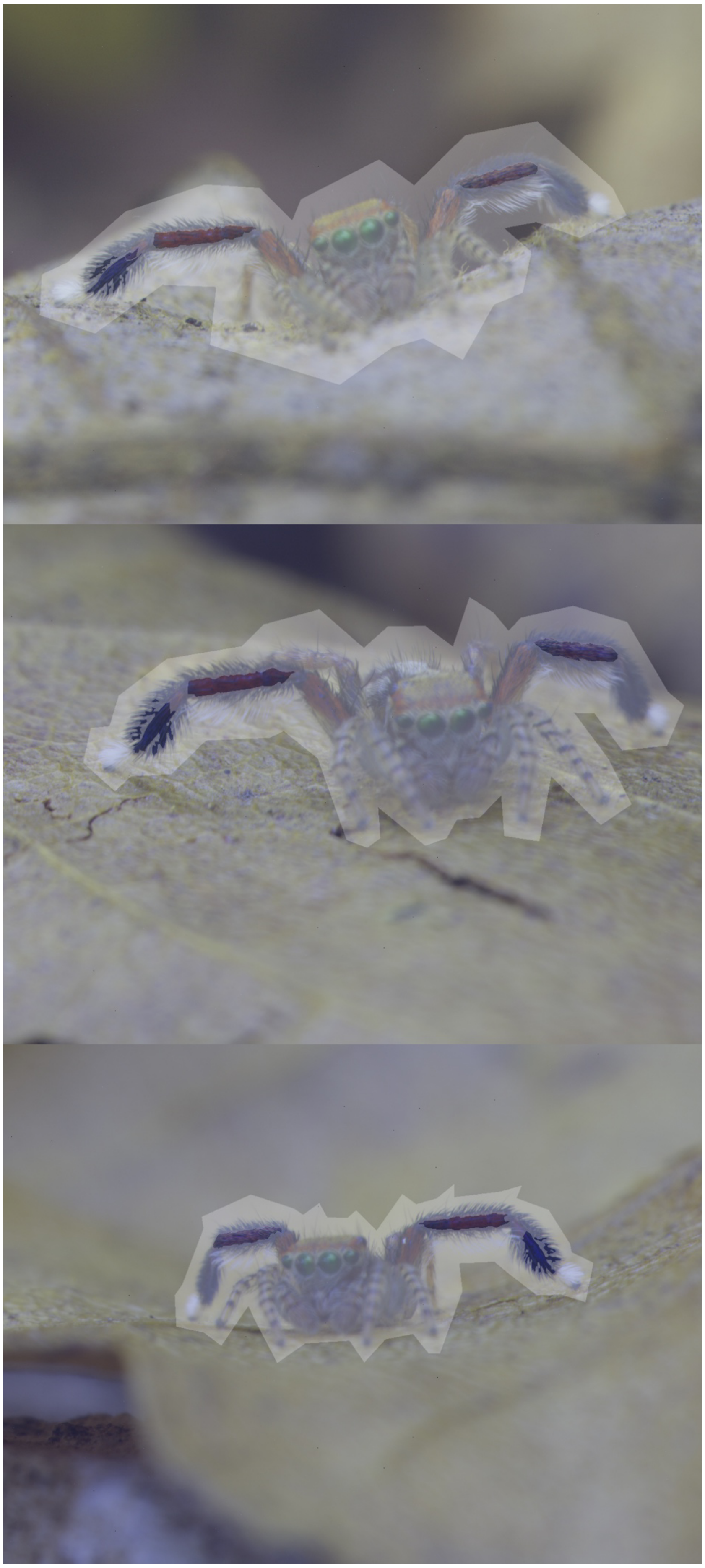

Supplementary Figure 1. Exemplar images showing a single spider photographed on three different leaves. Artificially lightened pixels correspond to regions not selected for analysis, whereas normally-exposed pixels correspond to the four regions selected for analysis: (1) black metatarsal cuticle + hairs, (2) perpendicularly-oriented red tibia + patella, (3) obliquely-oriented red (appearing orange or yellow) tibia + patella, and (4) background. Photos are false-color images with the receptor excitations of the avian LWS, MWS, and UVS cones plugged into the screen's red, green, and blue channels, respectively.

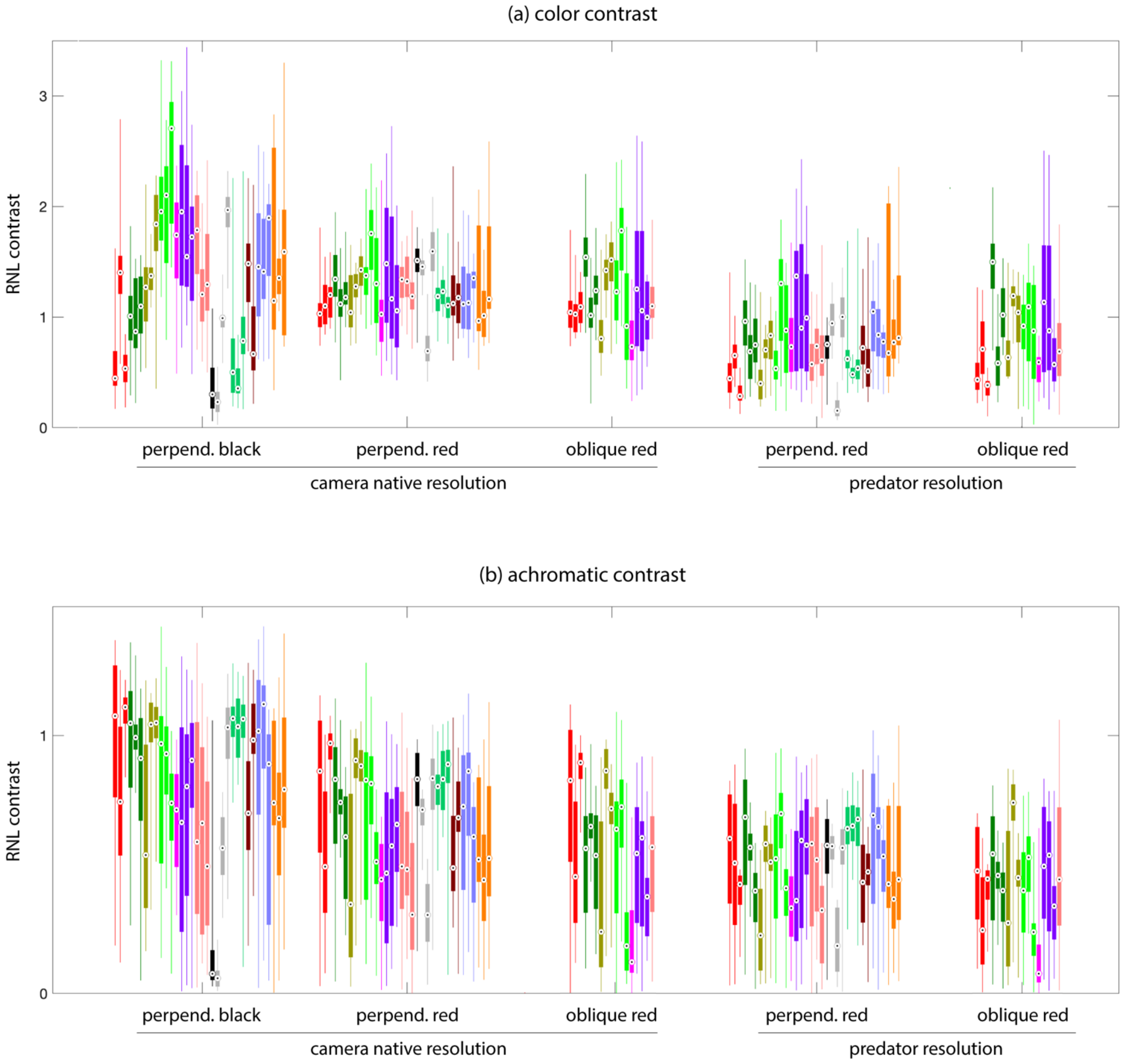

Supplementary Figure 2. More detailed versions of Figure 5a-b, showing (a) RNL color contrast and (b) RNL achromatic contrast with the background for different colors, orientations, and predator resolutions of male *S. barbipes*' ornamented legs. Summary boxplots of Figure 5 have been broken down into individual boxplots corresponding to every possible combination of spider and leaf ID. Boxplots of the same color correspond to the same individual spider.
